# Nozzle Pressure- and Screw Position-Based CAE Scientific Process Parameter Setup for Injection Molding Process

**DOI:** 10.3390/polym17020198

**Published:** 2025-01-14

**Authors:** Ren-Ho Tseng, Chien-Hung Wen, Chen-Hsiang Chang, Yu-Hao Chen, Chieh-Hsun Tsai, Sheng-Jye Hwang

**Affiliations:** Department of Mechanical Engineering, National Cheng Kung University, Tainan 701401, Taiwan

**Keywords:** injection molding, scientific process parameter setup, injection molding CAE simulation, nozzle pressure, screw position

## Abstract

This study developed a scientific process parameter setup based on nozzle pressure and screw position, with the process parameter search sequence being injection speed, *V*/*P* switchover position, packing pressure, and packing time. Unlike previous studies, this study focuses on the scientific process parameter setup of experiments and simulations, as well as on the implementation of calibration. Experiments and simulations had the same trend of results in the scientific process parameter setup. Although the experiments and simulations had the same trend, the machine response caused parameter errors. After setting the time constant of the simulations, injection speed profiles from the experiments and simulations became closely aligned. The simulation results for the injection speed and *V*/*P* switchover position became closer to the experiment results than the results of the uncalibrated simulation. The error between the simulated and experimental injection speed was reduced from 20% to 6% after applying time constant calibration. The *V*/*P* switchover point error was also reduced from 11% to 5%, highlighting the effectiveness of the time constant to calibrate the simulation.

## 1. Introduction

With the proliferation of Industry 4.0 concepts and the smart transformation of the injection-molding industry, T0 mass production is gradually becoming the goal pursued by the injection-molding industry. Mass production involves optimizing the mold design and processing parameters before proceeding to large-scale production. Trial 0 refers to the first trial molding conducted after the mold is completed, aimed at identifying and correcting issues in the mold and process. T0 mass production aims to enable large-scale production immediately after the first trial molding once the product design is finalized, significantly shortening both the production and trial molding cycles. Simulation and scientific process parameter setup are indispensable in achieving T0 mass production. Therefore, this study focuses on how to use experiment and simulation tools for scientific process parameter setup for T0 mass production.

To strengthen and refine the scientific process parameter setup, numerous scholars have dedicated considerable efforts to advancing this field. Su et al. [1] identified the appropriate *V*/*P* switchover position by locating the peak in the pressure curve and determined the optimal clamping force by analyzing the pressure differential. Liou et al. [2] identified the optimal *V*/*P* switchover position by locating the inflection point of the pressure peak, the injection speed by analyzing the pressure peak and the timing of the peak, the packing pressure by comparing product weight differences, and the clamping force by assessing variations in clamping force. Cheng et al. [3] identified the optimal injection speed by analyzing the pressure peak and the timing of the peak, determined the *V*/*P* switchover position by product weight, confirmed that no packing pressure was needed based on screw position and pressure fluctuations, and identified the suitable clamping force by evaluating clamping force differences. Nian et al. [4] defined a sharp pressure increase as the optimal *V*/*P* switchover position and determined the gate solidification time from pressure changes near the gate to set the packing time. Chang et al. [5,6] found that the early application of packing pressure affects the quality of parts farther from the gate and that multi-stage packing pressure results in a more uniform specific volume, reducing shrinkage and warping compared to single-stage packing. Huang et al. [7] found that a late *V*/*P* switchover position causes a pressure peak near the gate, while an early *V*/*P* switchover position leads to a decrease followed by an increase in the pressure curve. Reducing the final injection speed effectively minimizes warping, while excessively high first-stage packing pressure exacerbates warping and affects width dimensions. Leo et al. [8] found that insufficient packing time causes a sudden drop in cavity pressure, and single-stage packing pressure results in thicker areas farther from the gate. Chen et al. [9] proposed a method to determine gate solidification time by analyzing pressure–density relationships and real-time screw displacement. Research results show that a converging melt fill rate indicates that the gate has solidified. Fernandez et al. [10] found that the clamping force during filling and packing is relevant to the deformation of hybrid inserts of a mold. They used Moldex3D software to verify the results of deformation and cooling cycle time.

Due to the numerous factors affecting injection-molded product quality, a gap still exists between simulations and experiments. To address this, several scholars have proposed methods to improve simulation accuracy. Ahmad et al. [11] utilized the von Mises plasticity material model and boundary conditions to simulate tensile and flexural moduli of oil palm fiber composite and found that the simulation and experimental results matched well. Huang et al. [12,13] found that injection speed response is the primary factor causing discrepancies between simulations and experiments. Wang et al. [14] addressed factors causing discrepancies between simulations and experiments by analyzing screw position and pressure curves. Guerrier et al. [15] proposed incorporating nozzle units and screw acceleration into the simulation, considering that the melt compression behavior at the nozzle end during the simulation process effectively reduces the discrepancy in filling time between the simulation and the experiment. Regi et al. [16] found that simulations often overestimate injection pressure and fail to accurately represent the melt breaking through the solidified layer in thin-molded products. Vietri et al. [17] proposed incorporating the effects of pressure on viscosity and mold rebound into mold flow analysis, which helps make the pressure curve in simulations more closely match experimental results. Holger et al. [18] found that pressure’s first derivative shows a turning point where the melt encounters geometric changes. By comparing simulation and experimental pressure curves, the screw position at this turning point can be identified.

Based on the above factors, this study will install pressure sensors at the machine nozzle to monitor pressure throughout the injection-molding process. A procedure for searching process parameters will be proposed to guide the execution of scientific process parameter setup. The feasibility of this procedure will be validated through experiments and simulations, comparing the differences between simulation and experiment; identifying factors causing these differences, such as time constants; and making necessary corrections.

## 2. Theory and Background

### 2.1. Flow Characteristics of the Melt

**Figure 1:**
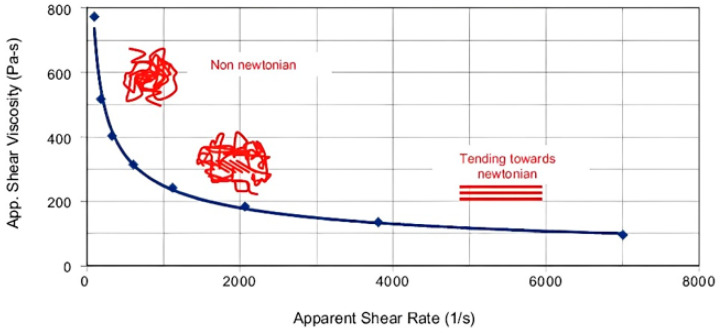
The effect of shear rate on viscosity.

In general, polymers are viscoelastic materials. During the injection-molding process, under high temperatures and shear rates, polymer melts behave more like viscous fluids. Thus, during the injection-molding process, plastic melt can be considered a fluid, with flow primarily driven by pressure and resistance to flow determined by viscosity. Most plastic melts are shear-thinning fluids. At low shear rates, polymer chains are typically entangled. When a higher shear rate is applied, these polymer chains align in the direction of flow and show a lower viscosity. As an even higher shear rate is applied, polymer chains align more in the flow direction, facilitating easier sliding of melt layers relative to one another and thus reducing flow resistance [19], and show an even lower viscosity. When the shear rate reaches a specific level and most molecular chains are no longer entangled, additional increases in shear rate lead to stabilized viscosity. Thus, polymer melt can be considered a fluid with instantaneous fixed viscosity values and can be considered a generalized Newtonian fluid, as shown in Figure 1.

The viscosity of melt shares the same unit as pressure multiplied by time, which allows the definition of a parameter to examine the relationship between shear rate and viscosity. In this context, the term “relative viscosity” is introduced, defined as the peak pressure multiplied by the time at which this peak occurs, to observe whether viscosity converges [20]. Since injection speed is proportional to the shear rate of the melt, adjusting the injection speed will also affect the shear rate during the injection process. If the relative viscosity shows minimal change during these adjustments, the change indicates that the relative viscosity has reached convergence [21]. This convergence suggests that the viscosity of the melt may have reached a state similar to that of a Newtonian fluid. Using such process parameters, where relative viscosity has converged, results in more stable product quality, even when environment or machine variations alter the shear rate during injection.

*Relative Viscosity* = *P_peak_* × *t_peak_* (1)

where *P_peak_* is the peak pressure and *t_peak_* is the time at which this peak pressure occurs. This study defines a convergence criterion for relative viscosity to determine when convergence occurs. If relative viscosity falls below this convergence value, the relative viscosity is confirmed to have converged.

*Vis_appropriate_* = *Vis_min_* + 0.05 × (*Vis_max_* − *Vis_min_*) (2)

where *Vis_appropriate_* is the relative viscosity value at convergence, *Vis_min_* is the minimum relative viscosity during the injection speed experiment, and *Vis_max_* is the maximum relative viscosity during the injection speed experiment.

### 2.2. Mold Flow-Filling Theory

This study employs Moldex3D 2023 R2RC, a mold flow analysis software. By leveraging advanced 3D mold flow technology, Moldex3D forecasts flow behavior, filling conditions, cooling effects, and potential defects and deformations in the plastic injection-molding process, effectively addressing flow issues in complex geometries. To ensure the precision of analysis results at every stage of the injection-molding process, Moldex3D applies the principles of fluid mechanics through the utilization of three fundamental equations: the continuity equation, momentum equation, and energy equation. These equations empower Moldex3D to conduct thorough mold flow-filling analysis, providing a robust framework for accurate evaluation and optimization.

(3) $$\frac{\partial \rho }{\partial t}+\nabla \cdot (\rho \overset{⃑}{V})=0$$

(4) $$\rho (\frac{\partial \overset{⃑}{V}}{\partial t}+\overset{⃑}{V}\cdot \nabla \overset{⃑}{V})=\nabla \;\cdot \vec{\vec{\sigma_{\mathrm{total}}}}+\rho \vec{g}$$

(5) $$\rho C_{p}\;(\frac{\partial T}{\partial t}+\overset{⃑}{V}\;\cdot \;\nabla T)=\nabla \;\cdot \;(k\nabla T)+(\vec{\vec{\tau }}\;:\nabla \vec{V})$$

where ρ is the density, t is the time, $\overset{⃑}{V}$ is the velocity vector, ∇ is divergence, $\vec{\vec{\sigma_{\mathrm{total}}}}$ is the total stress, $\vec{g}$ is gravity, $C_{p}$ is specific heat, T is the temperature, and k is the thermal conductivity.

### 2.3. Material Model

In this study, Moldex3D uses the modified Tait model for the *P*–*V*–*T* relationships. This model provides an accurate representation of the *P*–*V*–*T* relationships for semi-crystalline and amorphous materials and is widely used in CAE mold flow analysis [22].

(6) $$\hat{V}={\;\hat{V}}_{0}[1-C\ln(1+P/B)]+{\;\hat{V}}_{t}$$

(7) $${\hat{V}}_{0}=\left\{\begin{array}{l} b_{1S}+b_{2S}\bar{T},\;\mathrm{if}\;T\leq T_{t}\\ b_{1L}+b_{2L}\bar{T},\;\mathrm{if}\;T>T_{t} \end{array}\right.$$

(8) $$B=\left\{\;\begin{array}{l} b_{3S}\exp\left(-b_{4S}\bar{T}\right),\;\mathrm{if}\;T\leq T_{t}\\ b_{3L}\exp\left(-b_{4L}\bar{T}\right),\;\mathrm{if}\;T>T_{t} \end{array}\right.$$

(9) $$\bar{T}=T-b_{5}$$

(10) $${\;T}_{t}=b_{5}+b_{6}P$$

(11) $${\hat{V}}_{t}=\left\{\;\begin{array}{l} b_{7}\exp\left(b_{8}\bar{T}-b_{9}P\right),\;\mathrm{if}\;T\leq T_{t}\\ 0\;\;\;\;\;\;,\mathrm{if}\;T>T_{t} \end{array}\right.$$

(12) C=0.0894

where $\hat{V}$ represents the specific volume of the plastic material under specific conditions; ${\hat{V}}_{0}$ is the specific volume at zero pressure; $b_{1}$ and $b_{2}$ are parameters describing the temperature dependence of ${\hat{V}}_{0}$; *B* indicates the sensitivity to pressure and is a function of temperature, with material constants $b_{3}$ and $b_{4}$; $T_{t}$ is the transition temperature, with $b_{5}$ and $b_{6}$ describing how $T_{t}$ changes with pressure; and ${\hat{V}}_{t}$ is the specific volume altered by the crystallization behavior, with $b_{7}$, $b_{8}$, and $b_{9}$ describing specific parameters for semi-crystalline polymers during state transitions, while for amorphous materials, ${\hat{V}}_{t}$ is zero.

In this study, the Cross–William–Landel–Ferry (Cross–WLF) viscosity model is used in Moldex3D. This model is employed to describe how viscosity varies with temperature and shear rate in Newtonian and shear-thinning flow regions. This model’s ability to accurately approximate the conditions during the filling phase makes it one of the most commonly used models for characterizing polymer viscosity [23].

(13) $$\eta =\frac{\eta_{0}}{1+(\frac{\eta_{0}\dot{\gamma }}{\tau^{*}}{)}^{1-n}}$$

(14) $$\eta_{0}=D_{1}\exp\left(\frac{-A_{1}\left(T-T_{c}\right)}{A_{2}+\left(T-T_{c}\right)}\right)$$

(15) $$T_{c}=D_{2}+D_{3}P$$

(16) $$A_{2}={\tilde{A}}_{2}+D_{3}P$$

where η represents the melt viscosity; $\eta_{0}$ is the viscosity at zero shear rate; $\dot{\gamma }$ is the shear rate; n is the power-law index; $\tau^{*}$ is the critical shear stress; $T_{c}$ is the glass transition temperature, which is dependent on pressure; $D_{1}$ is a model constant describing the viscosity at zero shear rate, at the glass transition temperature, and under atmospheric pressure; $D_{2}$ is a model constant describing the glass transition temperature under atmospheric pressure; $D_{3}$ is a model constant representing the change in glass transition temperature with pressure, often considered zero, as pressure’s impact on viscosity is usually negligible; $A_{1}$ is a model constant showing the temperature dependence of the glass transition temperature under zero shear rate conditions; and $A_{2}$ is a constant depending on the material type considered.

### 2.4. First-Order Linear Regression

First-order linear regression is a statistical method used to analyze the relationship between an independent variable (the cause) and a dependent variable (the effect). The independent variable affects the dependent variable but is not influenced by the dependent variable.

(17) $$Y=\beta_{0}\;+\beta_{1}\times X$$

where *Y* is the dependent variable, *X* is the independent variable, $\beta_{1}$ is the slope representing the change in *Y* for each unit change in *X*, and $\beta_{0}$ is the intercept representing the predicted value of *Y* when *X* is zero. The goal of first-order linear regression is to estimate $\beta_{1}$ and $\beta_{0}$ so that the regression line best fits the data. This is typically achieved using the method of least squares, which minimizes the sum of the squared vertical distances from all points to the regression line.

When evaluating model performance, *R*^2^ measures how well the independent variable explains the variation in the dependent variable, ranging from 0 to 1, with values closer to 1 indicating a better fit. In a first-order linear regression model, *R*^2^ reflects the model’s goodness of fit.

(18) $$R^{2}=1-\frac{\sum {\left(y_{i}-\hat{y_{i}}\right)}^{2}}{\sum {\left(y_{i}-\overset{-}{y}\right)}^{2}}$$

where $y_{i}$ is the actual value of the dependent variable, $\hat{y_{i}}$ is the predicted value of the dependent variable from the model, and $\overset{-}{y}$ is the mean value.

## 3. Experimental Setup

### 3.1. Equipment

The injection-molding machine used in this study is a 300-ton hydraulic model HT-300, manufactured by Fu Chun Shin Machinery Manufacture Co., Ltd (Tainan, Taiwan). The HT-300 has a 54 mm screw diameter, a maximum injection pressure of 244.87 MPa, and a maximum injection speed of 160 mm/s. The machine supports the OPC UA communication protocol, facilitating seamless data transmission between the computer and the machine.

The measurement device used in this study is a 2.5D image measuring instrument from Zong-Sing Machinery Co., Ltd. (Kaohsiung, Taiwan), model ZS-4030. The measurement device offers X/Y/Z travel ranges of 400 × 300 × 200 mm and a resolution of 0.001 mm.

### 3.2. Material

The experiment material used in this study is acrylonitrile styrene (SAN), model PN-107 L125FG, produced by Chi Mei Corporation (Tainan, Taiwan). This material has a melt index of 58 mL/10 min, a density of 1.06 g/cc, and a shrinkage rate of 0.2–0.7%.

### 3.3. Data Acquisition System

**Figure 2:**
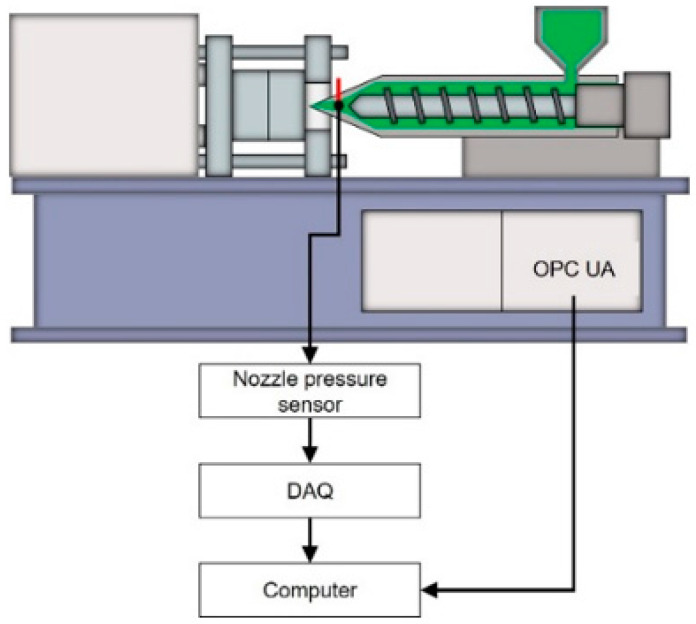
Data acquisition system architecture.

This study involves capturing data from pressure sensors via a DAQ system and retrieving data from in-machine sensors using the OPC UA communication protocol. The architecture of the data acquisition system is shown in Figure 2. This study utilizes a pressure sensor produced by Dynisco (Franklin, MA, USA), model PT4656XL-30M, which has a pressure measurement range of 0–30,000 psi and can withstand temperatures of up to 400 °C. The DAQ used is from Advantech Co., Ltd. (Taipei, Taiwan), model USB-4716, with a maximum sampling rate of 200 kHz.

## 4. Simulation Setup

### 4.1. Geometry

**Figure 3:**
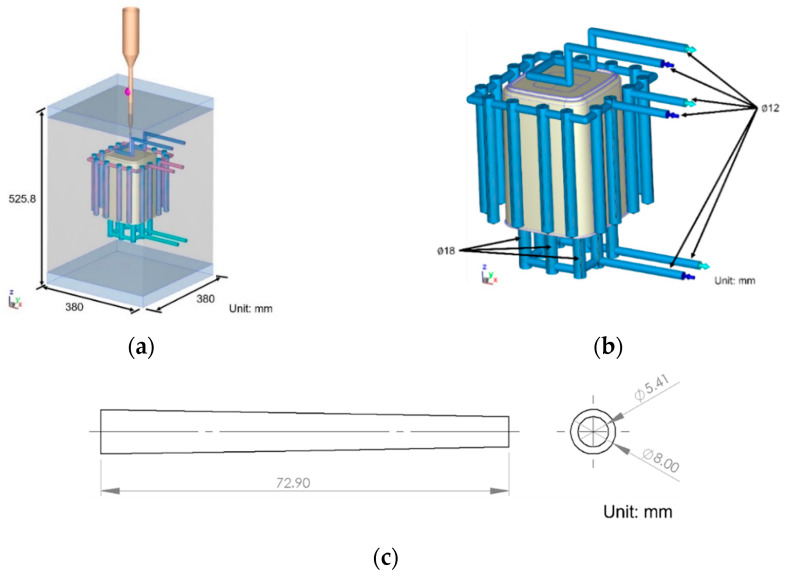
Geometric dimensions: (**a**) mold; (**b**) cooling channel; (**c**) runner.

**Figure 4:**
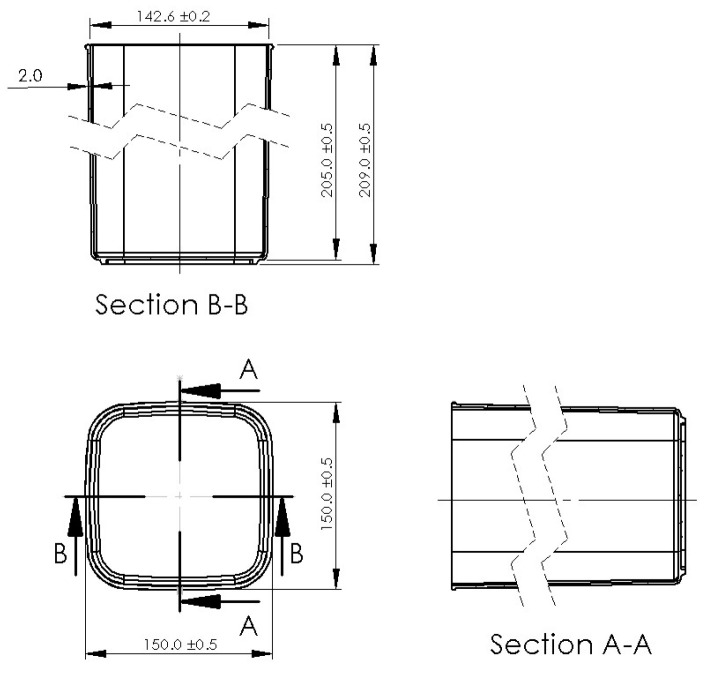
Part dimensions.

**Figure 5:**
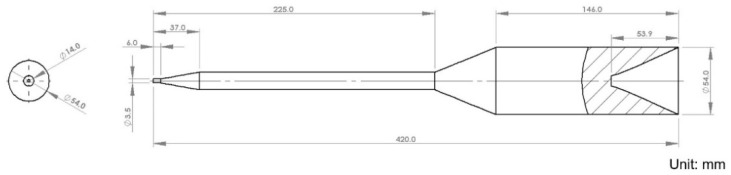
Nozzle zone dimensions.

This study uses a single-cavity cold runner mold produced by Felli Co., Ltd. (Tainan, Taiwan) for both experiments and simulations, primarily utilizing a sprue gate for filling. Measurements focus on the product’s inner diameter, which has a specified dimension of 142.4 mm. The model in this study includes the mold, cooling channels, runner, part, and nozzle area, with dimensions shown in Figure 3, Figure 4 and Figure 5.

### 4.2. Simulation Setting

**Figure 6:**
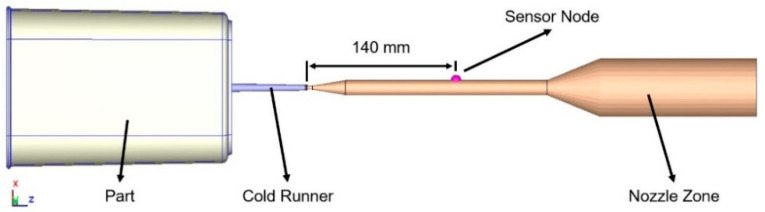
Simulation sensor node location.

In this study, the sensor nodes are positioned 140 mm from the sprue point to record the pressure history at the nozzle end, as shown in Figure 6.

In this study, 5 layers of boundary layer mesh are set up on both the top and bottom of the thickness direction, with 2 mm tetrahedral mesh used for growth at the center of the thickness. The volume and number of meshes in the model is shown in Table 1.

## 5. Results and Discussion

### 5.1. Experimental Results

**Figure 7:**
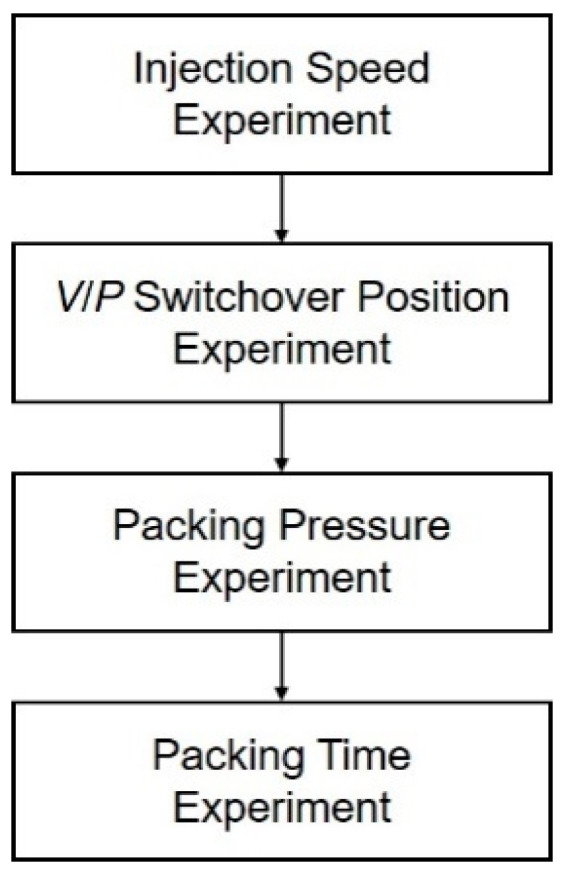
Process parameter search flow.

In the scientific process parameter setup, parameters from later stages have less impact on earlier stages. Therefore, this study defines the scientific process parameter setup flow based on the sequential order of stages in the injection-molding process. Injection speed, which affects viscosity and subsequently the quality of the filling stage, is the first parameter to be selected. The *V*/*P* switchover position, which influences the material volume during injection, is chosen next. Both packing pressure and packing time significantly impact the results of the packing stage. While packing time is often determined by the gate solidification time, packing pressure affects this solidification time. Thus, packing pressure is selected first, followed by the determination of the appropriate packing time. The process parameter search flow is shown in Figure 7.

#### 5.1.1. Injection Speed Experiment

As the injection speed increases, the shear rate correspondingly rises. When the shear rate reaches a specific threshold, the melt behaves like a Newtonian fluid, exhibiting minimal changes in viscosity with variations in shear rate. This results in improved stability of the product quality. This study introduces the concept of relative viscosity and uses different injection speeds to identify the speed at which relative viscosity converges.

Since the relative viscosity in the injection speed experiment is based on peak pressure, and to avoid the influence of the rapid pressure increase that occurs after the mold cavity is filled on the calculated relative viscosity, a *V*/*P* switchover position where the mold cavity is not fully filled is selected for the experiment. To ensure that the product can be smoothly ejected by the mold’s ejection mechanism during production, a portion of the packing pressure is applied in this experiment to ensure that the cavity is fully filled throughout the process. The parameters used in the injection speed experiment are shown in Table 2.

(19) $$V/P_{\mathrm{initial}}=\mathrm{Positio}n_{\mathrm{storage}\;}-0.95\;\times \;(\frac{V_{\mathrm{Runner}\;}+V_{\mathrm{Product}}}{\frac{\pi }{4}\;\times \;D_{\mathrm{screw}}^{2}})$$

where $V/P_{\mathrm{initial}}$ is the initial *V*/*P* switchover position, $\mathrm{Positio}n_{\mathrm{storage}}$ is the screw position at the end of storage, $V_{\mathrm{Runner}}$ is the runner volume, $V_{\mathrm{Product}}$ is the product volume, and *D_screw_* is the screw diameter.

**Figure 8:**
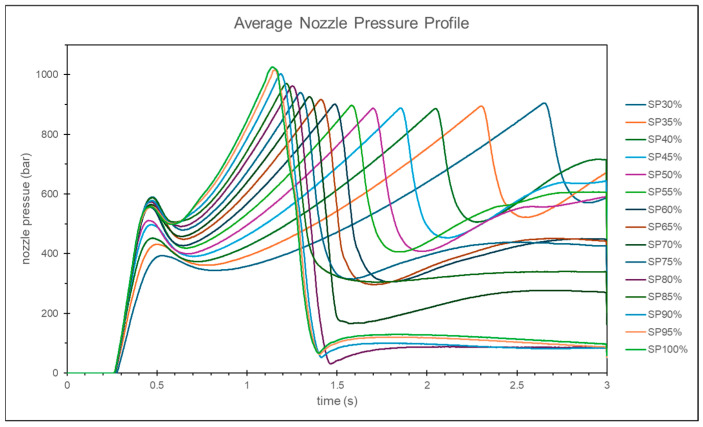
Experimental nozzle pressure curves at different injection speeds.

At lower injection speeds, the nozzle peak pressure tends to remain constant, while the change in the timing of the peak pressure is more significant. Conversely, at higher injection speeds, the nozzle peak pressure shows larger variations, but the change in the timing of the peak pressure is relatively small, as shown in Figure 8.

**Figure 9:**
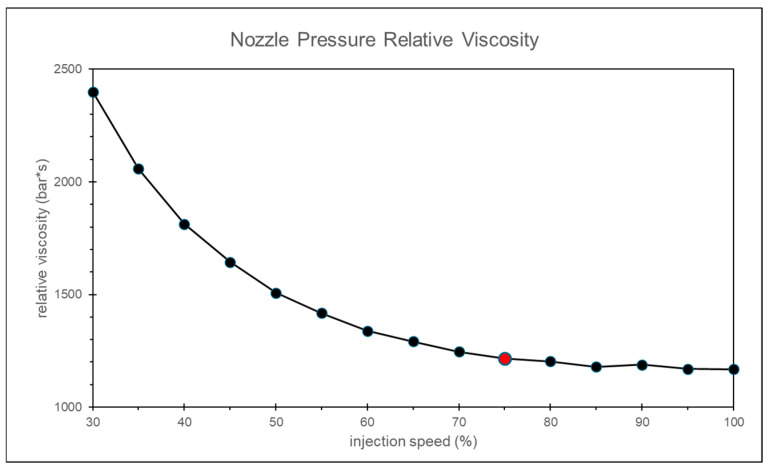
Experiment of relative viscosity at different injection speeds.

By calculating the relative viscosity using the nozzle peak pressure and the timing of the peak pressure, and applying the convergence criterion, the most appropriate injection speed is found to be 75% of the maximum injection speed, as shown in Figure 9.

#### 5.1.2. *V*/*P* Switchover Position Experiment

The *V*/*P* switchover position is the setting that determines the screw position at which the machine transitions from velocity control to pressure control. An early *V*/*P* switchover may cause short shots, while a late switchover can lead to mold overpacking. In this study, the screw position is determined by analyzing the second derivative of the pressure curve, which indicates when the cavity is fully filled. The initial *V*/*P* switchover position for this experiment is primarily determined based on the calculation of the cavity filling volume. The experiment process parameters are shown in Table 3.

(20) $$V/P_{\mathrm{initial}}=\mathrm{Positio}n_{\mathrm{storage}\;}-(\frac{V_{\mathrm{Runner}\;}+V_{\mathrm{Product}}}{\frac{\pi }{4}\;\times \;D_{\mathrm{screw}}^{2}})$$

**Figure 10:**
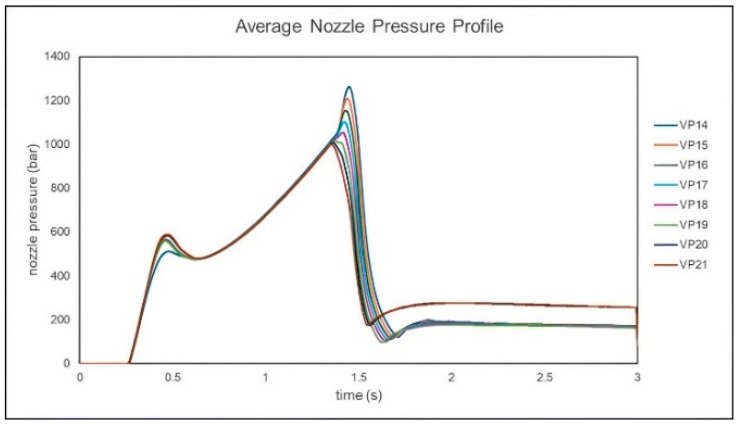
Experimental nozzle pressure curves for different *V*/*P* switchover positions.

**Figure 11:**
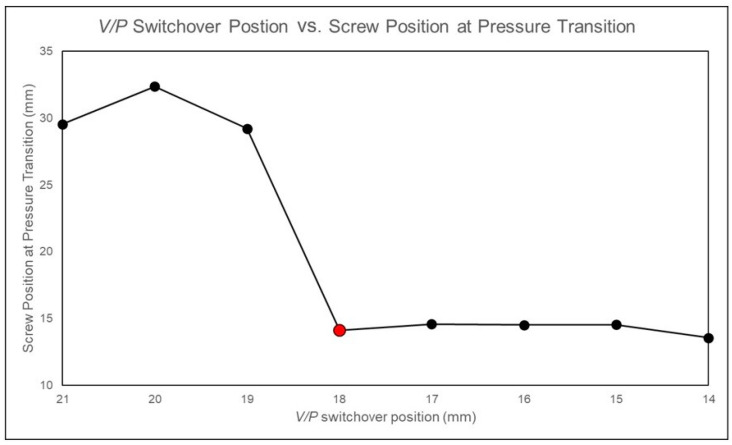
Experimental screw positions for fully filling the cavity identified at different *V*/*P* switchover positions.

When the *V*/*P* switchover position setting is below 18 mm, the nozzle pressure curve exhibits a rapid increase, as shown in Figure 10. By performing a second derivative on the nozzle pressure curve and identifying the screw position corresponding to the local maximum in this derivative, the screw position where the cavity is fully filled is determined and recorded, as shown in Figure 11. Observations show that for *V*/*P* switchover position settings below 18 mm, the determined screw positions are consistent. To ensure the cavity is not fully filled before entering the packing stage, a *V*/*P* switch position of 19 mm is determined to be the optimal process parameter.

#### 5.1.3. Packing Pressure Experiment

The magnitude of the packing pressure determines the amount of melt compensation entering the cavity during the packing stage, which significantly affects the dimensions and appearance of the product. Therefore, this study measures the product dimensions and determines the appropriate packing pressure based on the product dimensions. The process parameters for the packing pressure experiment are shown in Table 4.

**Figure 12:**
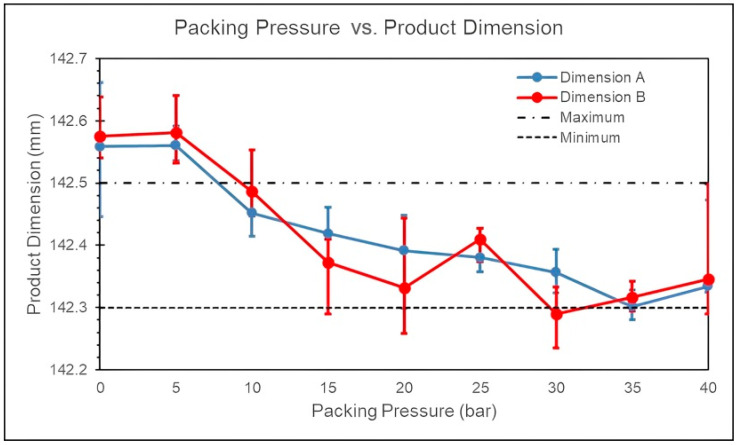
Experiment of product dimensions for different packing pressures.

The specified dimension of the product in this study is 142.4 mm. When the packing pressure is set to 25 bar, the product dimensions are closest to the specified dimensions, as shown in Figure 12. This parameter setting provides a larger molding window, indicating that a packing pressure of 25 bar is the close to the optimal process parameter.

#### 5.1.4. Packing Time Experiment

**Figure 13:**
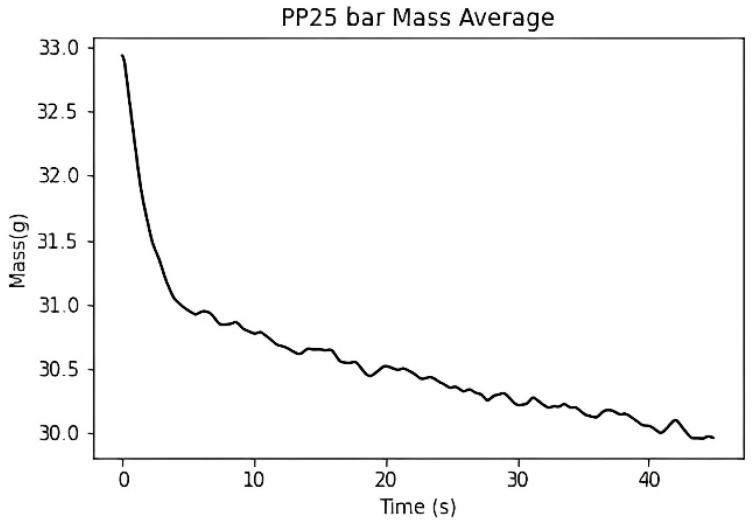
Experimental melt mass at the front end of the screw.

The duration of the packing pressure stage is often determined by the gate solidification time. Once the gate solidifies, the melt can no longer continue filling the cavity. Assuming no material leakage occurs at the check valve, it can be inferred that the melt mass difference at the front end of the screw will show a converging trend. Therefore, in this study, the temperature and pressure at the machine nozzle are applied to the material’s *P*–*V*–*T* model to calculate the specific volume. The screw front volume is obtained by multiplying the screw position by the cross-sectional area and then dividing the screw front volume by the material’s specific volume to calculate the melt mass at the front end of the screw, as shown in Figure 13.

**Figure 14:**
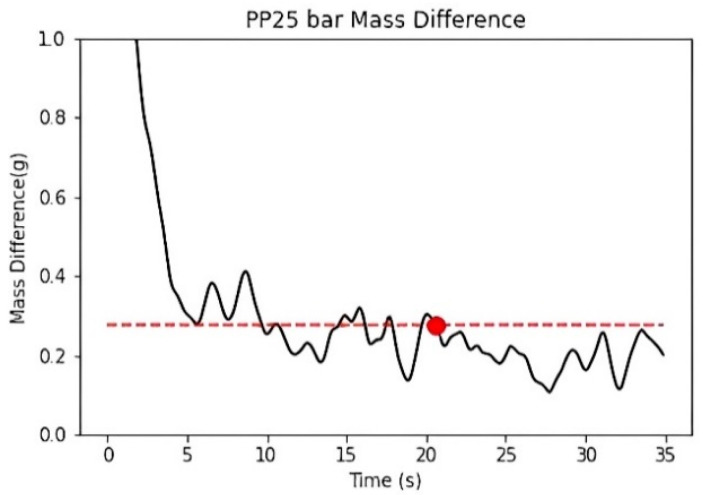
Experiment of melt mass difference at the front end of the screw. (dashed line: 0.1% of the ideal product weight).

To determine the appropriate packing pressure time, the melt mass is subtracted from the melt mass 10 s later to obtain the melt mass change. The packing time is defined as appropriate if the melt mass difference within the 10 s is less than 0.1% of the ideal product weight. The final packing time, determined through the convergence criterion, is approximately 21 s, as shown in Figure 14.

In this study, to verify the feasibility of using this method to determine the packing time, a search is conducted around the identified packing time. The convergence trend is then examined by measuring the product weight. The process parameters for the packing time experiment are shown in Table 5.

**Figure 15:**
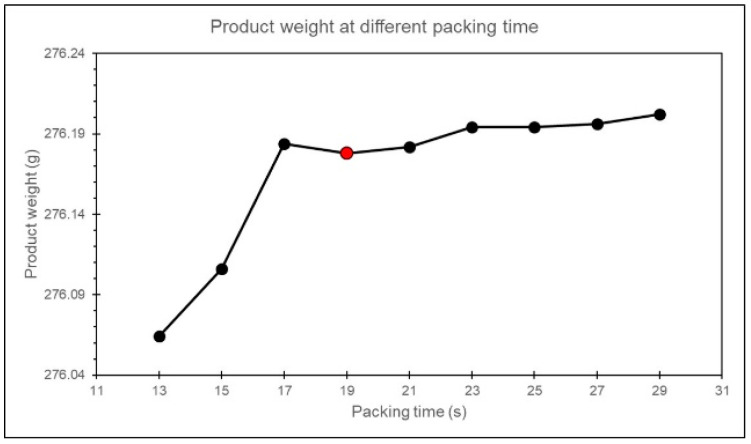
Experiment of product weight with different packing time.

In this study, it is observed that when the packing time is set to 19 s, the product weight exhibits a converging trend, confirming the feasibility of this method, as shown in Figure 15.

### 5.2. Simulation Results

In this study, to verify whether this scientific process parameter setup can be applied to simulation, the same scientific process parameter setup is incorporated into the simulation and compared with the experimental results. Finally, adjustments are made based on the machine response of the simulation.

#### 5.2.1. Injection Speed Simulation

In the injection speed simulation, the process parameters and convergence criteria used are the same as those in the injection speed experiment. The process parameters are shown in Table 6.

**Figure 16:**
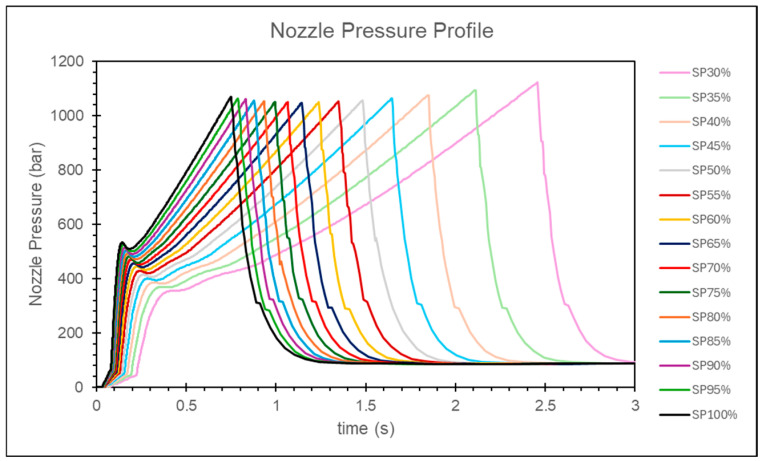
Simulation of nozzle pressure curves at different injection speeds.

**Figure 17:**
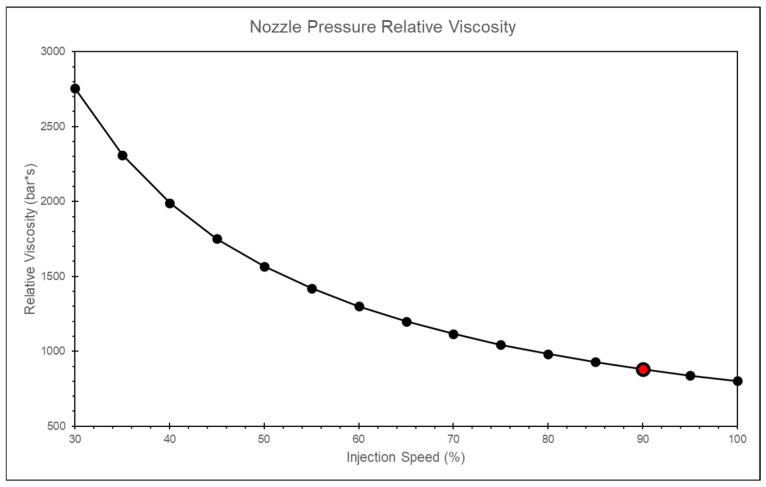
Simulation of relative viscosity at different injection speeds.

In this study, it is observed that as the injection speed increases, the change in the peak pressure is relatively small. However, the variation in the timing of the peak pressure is larger at lower injection speeds and smaller at higher injection speeds, as shown in Figure 16. By calculating the relative viscosity using the peak pressure and the timing of the peak pressure, and applying the convergence criteria, the optimal injection speed is determined to be 90% of the maximum injection speed, as shown in Figure 17.

**Figure 18:**
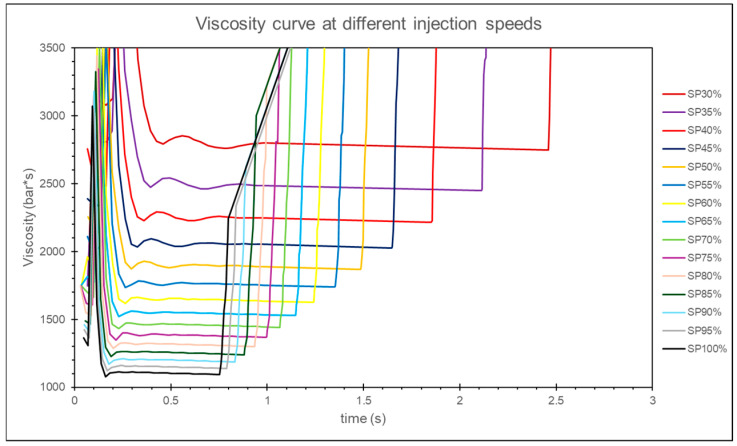
Simulation of melt viscosity curves for different injection speeds.

**Figure 19:**
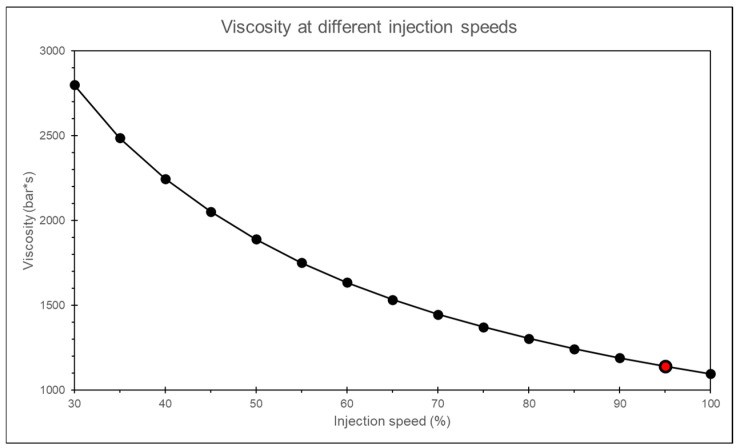
Simulation of melt viscosity at different injection speeds.

By observing the melt viscosity at the sensor node position, the results show that after a period of filling during the injection stage, the melt viscosity stabilizes at a constant value, as shown in Figure 18. Therefore, this study captures the melt viscosity at the 90% filling time point for different injection speeds and compares the viscosity for various injection speeds, as shown in Figure 19.

**Figure 20:**
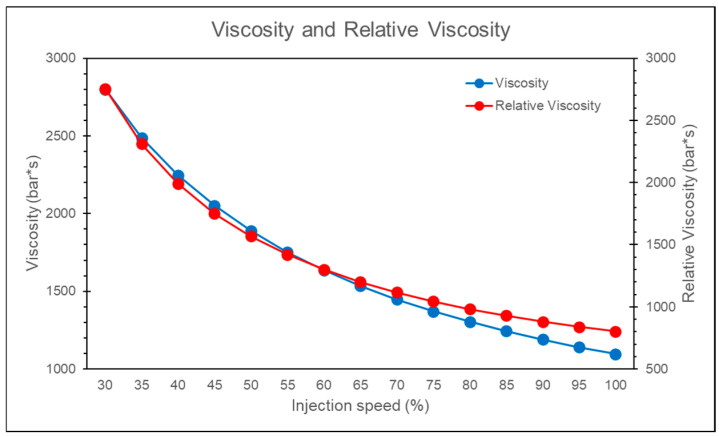
Comparison chart of relative viscosity and melt viscosity.

Overlaying the relative viscosity with the melt viscosity for different injection speeds reveals that both exhibit similar trends, as shown in Figure 20. The simulation results confirm that using the convergence of relative viscosity is an effective method for identifying the injection speed at which the melt viscosity is more stable.

#### 5.2.2. *V*/*P* Switchover Position Simulation

In the *V*/*P* switchover position simulation, except for the injection speed, which uses the process parameters identified in the injection speed simulation, all other process parameters are the same as those used in the *V*/*P* switchover position experiment. The process parameters for the *V*/*P* switchover position simulation are shown in Table 7.

**Figure 21:**
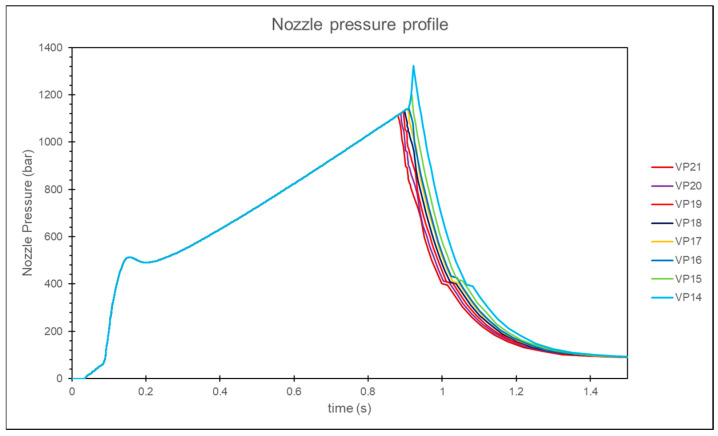
Simulation of nozzle pressure curves for different *V*/*P* switchover positions.

**Figure 22:**
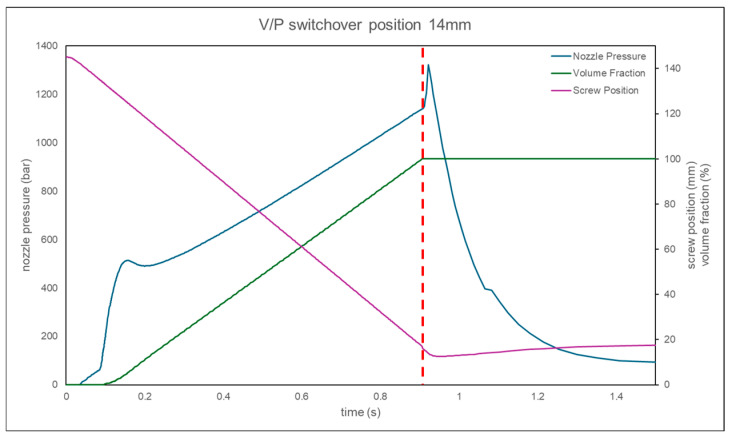
Simulation of the curve for a *V*/*P* switchover position of 14 mm.

When the *V*/*P* switchover position is set to less than 15 mm, the nozzle pressure curve shows a rapid increase, indicating that the melt has filled the cavity. Therefore, the optimal *V*/*P* switchover position is chosen to be 16 mm, as shown in Figure 21. At the same time, comparing the nozzle pressure curve, screw position, and volume filling percentage results for a *V*/*P* switchover position of 14 mm, the comparison reveals that the inflection point corresponds to a volume filling percentage of approximately 100%, and the corresponding screw position is about 15.8 mm, as shown in Figure 22. This study confirms the feasibility of using the inflection point to identify the screw position where the cavity is fully filled.

#### 5.2.3. Packing Pressure Simulation

In the packing pressure simulation, the process parameters continue from the results of the *V*/*P* switchover position simulation. For the packing pressure simulation, the same parameters as those used in the packing pressure experiments are chosen. The process parameters for the packing pressure simulation are shown in Table 8.

**Figure 23:**
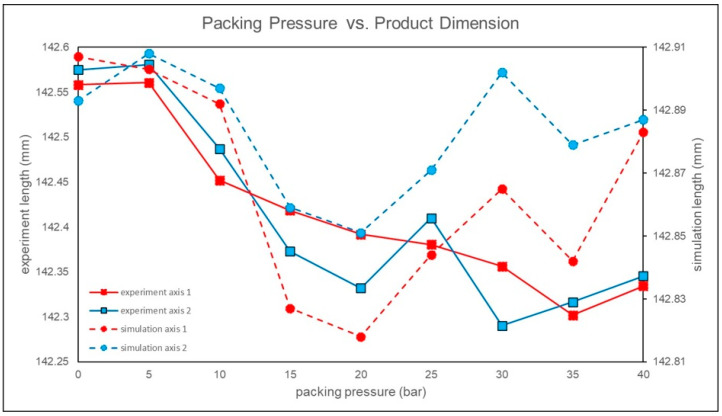
Product dimensions for different packing pressures in experiments and simulations.

Axis 1 refers to the dimension parallel to the direction of gravity, while Axis 2 refers to the dimension perpendicular to the direction of gravity, as shown in Figure 23. In this study, it can be observed that the simulated packing pressure shows the same trend as the experiment along Axis 2, whereas there is a significant difference in trends along Axis 1. Since the simulation does not yield results within the specified dimensions of 142.4 mm, a packing pressure of 25 bar, which is closer to the specified dimensions, is chosen for subsequent simulations of the packing pressure time.

#### 5.2.4. Packing Time Simulation

**Figure 24:**
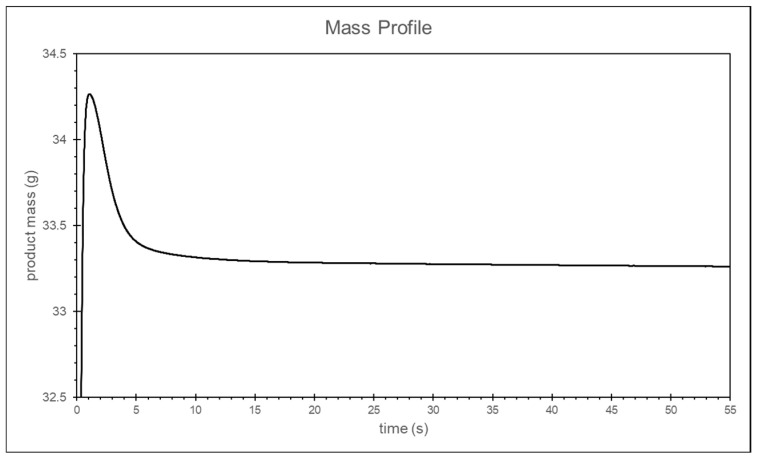
Simulation of the melt mass at the front end of the screw.

**Figure 25:**
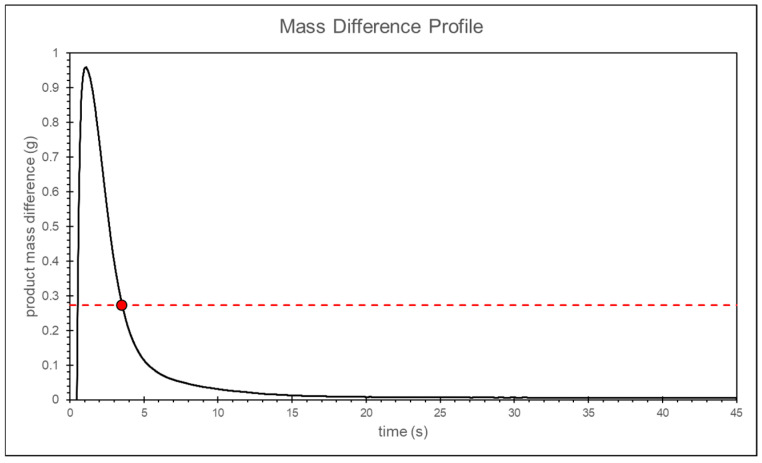
Simulation of the melt mass difference at the front end of the screw.

In the simulation of packing time, the same method used in the packing time experiment to calculate the melt mass difference and the convergence criteria is applied. This calculation is specifically conducted for the results at a packing pressure of 25 bar, as shown in Figure 24 and Figure 25. Ultimately, the appropriate packing time is determined to be around 4 s using the convergence criterion.

**Figure 26:**
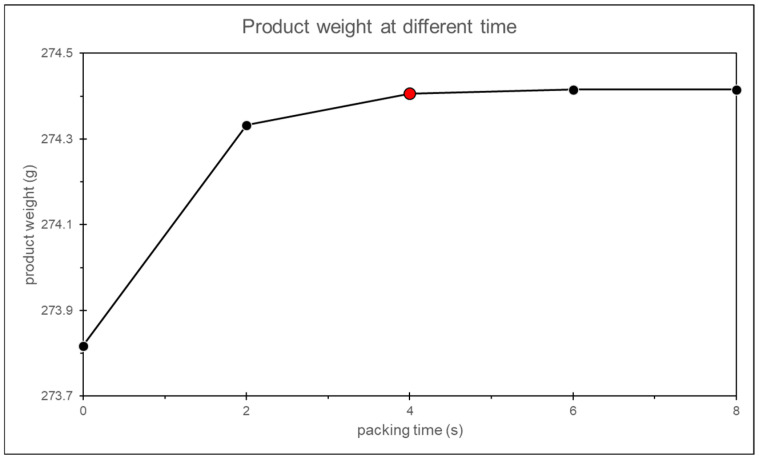
Simulation of the product weight with different packing time.

To verify whether the above method is feasible within the simulation, a search around the identified packing time is conducted, and the product weight at different packing times is recorded to confirm whether the gate solidified. In this study, it is observed that the product weight converges at a packing time of 4 s, confirming that this method is applicable in both experiments and simulations, as shown in Figure 26.

#### 5.2.5. Injection Speed Simulation Calibration

Huang et al. [12,13] identified that the machine’s response is one of the reasons for the discrepancies between the simulation and the results. In Moldex3D, the injection speed time constant represents the machine’s response speed and is defined as the time required for the injection speed to reach 80% of the set value under ideal assumptions. Since Moldex3D considers the machine’s resistance during the calculation of the injection speed time constant, half of the time required to reach 80% of the injection speed must be entered into Moldex3D.

**Figure 27:**
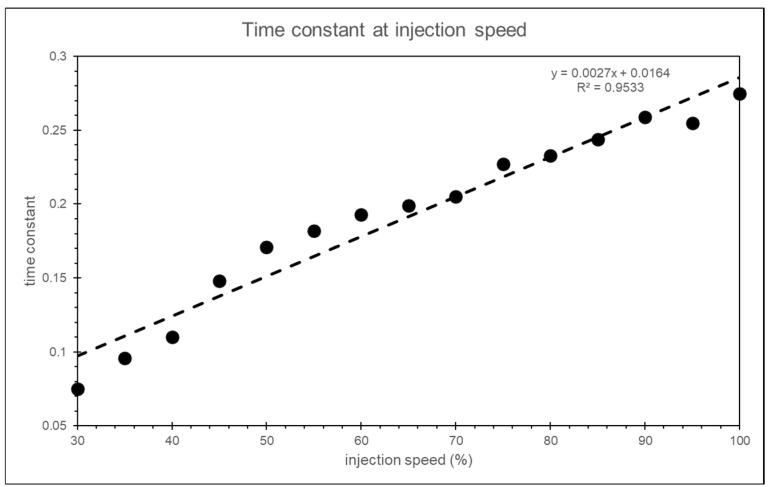
Time constants for different injection speeds.

*Time Constant* = *t*_0.8×*injection_speed*_/2 (21)

where *t*_0.8×*injection_speed*_ is the time from the start of injection to the point where the injection speed reaches 80% of the set value. In this study, the time constant for each injection speed is determined using the above formula, and the time constant for the input into the simulation is obtained through linear regression, as shown in Figure 27.

**Figure 28:**
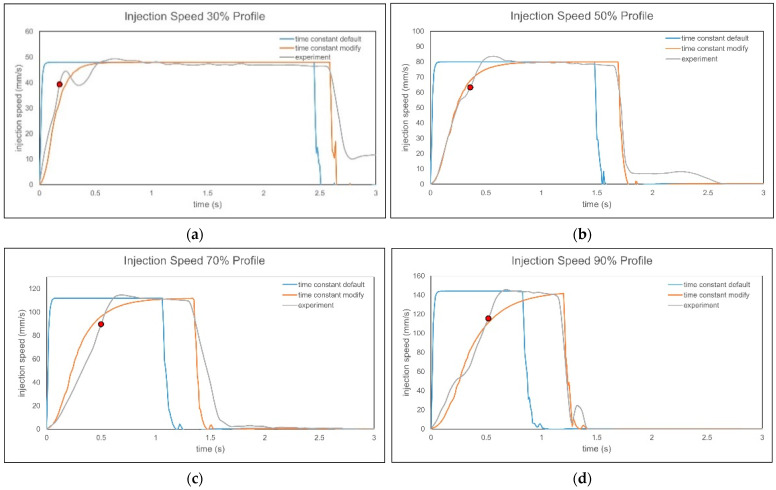
Simulation and experiment of injection speed curves before and after calibration for (**a**) 30%, (**b**) 50%, (**c**) 70%, and (**d**) 90%.

By comparing the injection speed curves before and after time constant correction, the results show that at lower injection speeds, the experiment and simulation injection speed curves are very close after correction. However, at higher injection speeds, differences remain between the experiment and the simulation due to the different methods of achieving the target injection speed, as shown in Figure 28. In the picture, the red dots indicate the points where 80% of the injection speed is reached, demonstrating that the calculated positions using the above formula are highly accurate and confirming the effectiveness of the method.

**Figure 29:**
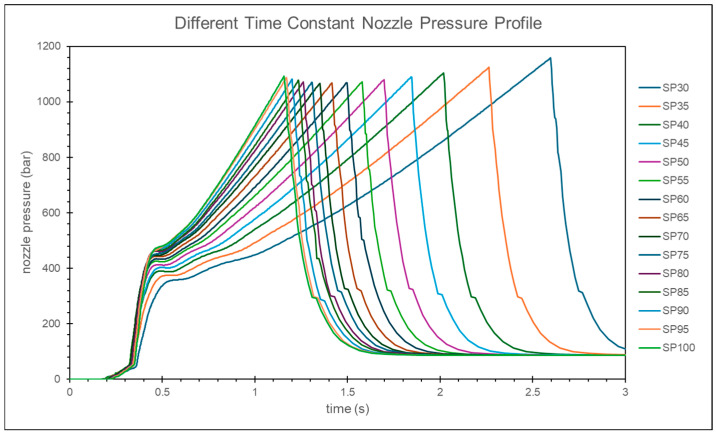
Nozzle pressure curves after adjusting the time constant.

**Figure 30:**
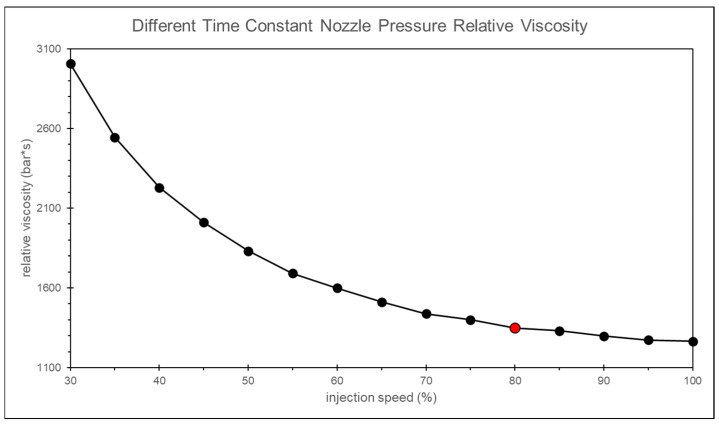
Relative viscosity curves after adjusting the time constant.

The timing of the nozzle peak pressure in the calibrated simulation aligns more closely with the actual experiment, as shown in Figure 29. Additionally, by analyzing the trend of relative viscosity, the optimal injection speed is found to be 80%, which is closer to the experiment results than the uncalibrated scenario, as shown in Figure 30.

**Figure 31:**
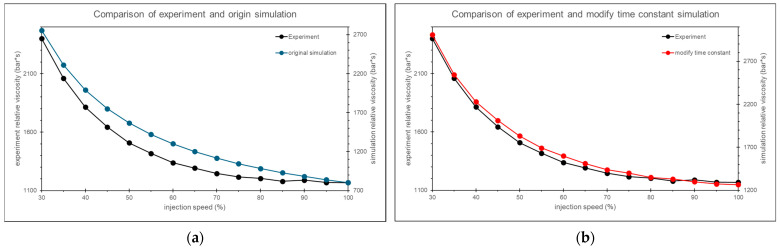
Comparison of relative viscosity curves between the experiment and the simulation (**a**) before calibration and (**b**) after calibration.

By comparing the relative viscosity curves from the simulation before and after calibration with the experimental relative viscosity curve, the results show that the calibrated simulation more closely aligns with the experimental trend, as shown in Figure 31.

#### 5.2.6. *V*/*P* Switchover Position Simulation Calibration

In the *V*/*P* switchover position simulation calibration, except for the injection speed and time constant, which uses the process parameters identified in the injection speed simulation calibration, all other process parameters are the same as those used in the *V*/*P* switchover position experiment. The process parameters for the *V*/*P* switchover position simulation calibration are shown in Table 9.

**Figure 32:**
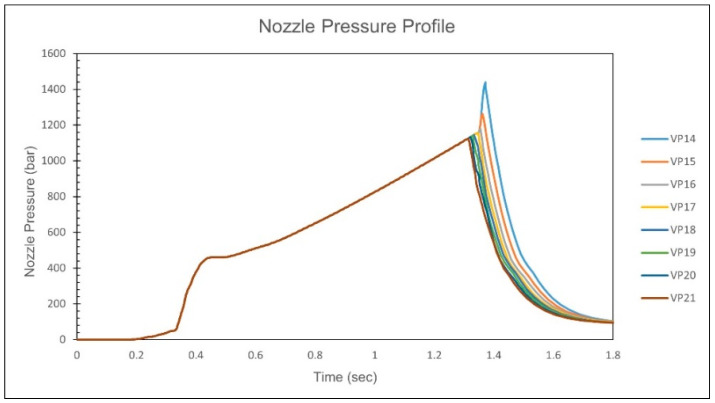
Simulation of calibration nozzle pressure curves for different *V*/*P* switchover positions.

**Figure 33:**
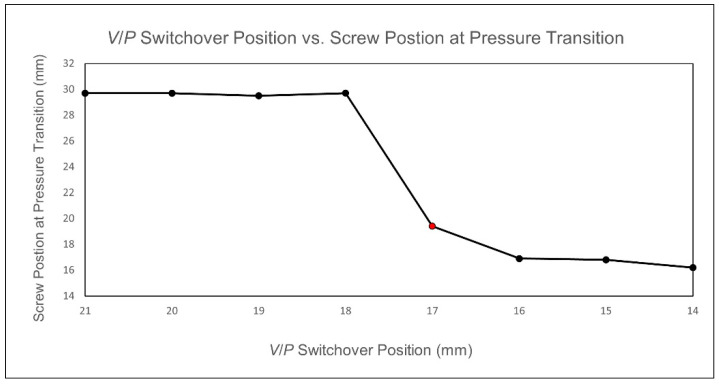
Simulation of calibration screw positions for fully filling the cavity identified at different *V*/*P* switchover positions.

**Figure 34:**
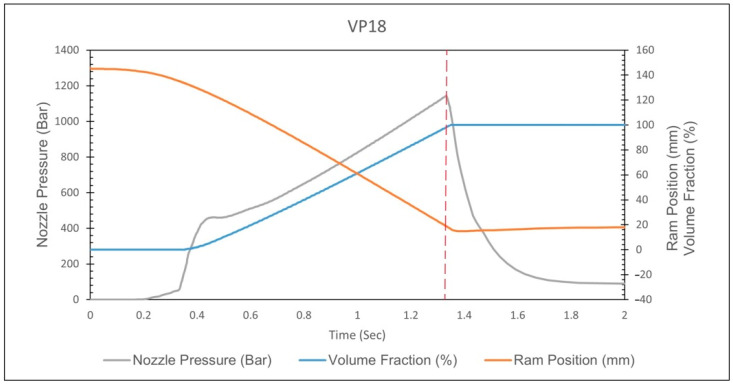
The nozzle pressure, ram position, and volume fraction at the *V*/*P* switchover position of 18 mm.

When the *V*/*P* switchover position setting is below 17 mm, the nozzle pressure curve exhibits a rapid increase, as shown in Figure 32. By performing a second derivative on the nozzle pressure curve and identifying the screw position corresponding to the local maximum in this derivative, the screw position where the cavity is fully filled is determined and recorded, as shown in Figure 33. It can be observed that for *V*/*P* switchover position settings below 17 mm, the determined screw positions are consistent. To ensure the cavity is not fully filled before entering the packing stage, the *V*/*P* switchover position of 18 mm is ultimately determined to be the optimal process parameter. At the same time, the volumetric fraction at each *V*/*P* switchover position is also checked, as shown in Table 10. The results show that at a *V*/*P* switchover position of 14 to 17 mm, the volumetric fraction exceeds 95%, turning to the compression phase. By comparing the volumetric fraction at the *V*/*P* switchover position of 18 mm with the nozzle pressure peak and screw position, it is found that the volumetric fraction at the *V*/*P* switchover position of 18 mm corresponds to approximately 88.57% of the nozzle pressure peak, consistent with the previous statement that the compression phase has not yet started, as shown in Figure 34. Therefore, the *V*/*P* switch position of 18 mm is chosen as the most appropriate. After the simulation calibration of the injection speed and *V*/*P* switchover position, the injection speed and *V*/*P* switchover position from the simulation become increasingly close to the experiment, proving the effectiveness of the time constant.

Tasello et al. [24] identified that inconsistencies and uncertainties in the experimental data must be minimized to prevent the introduction of uncertainties into the simulation calculations. To reduce the gap between experiments and simulations, Huang et al. [12,13] calibrated the injection response, and the deviation difference between simulations and experiments was reduced.

This study reduces the percentage error compared to the experiments by adjusting the time constant in Moldex3D. The error between the simulated and experimental injection speed is reduced from 20% to 6% after applying time constant calibration. The *V*/*P* switchover point error is also reduced from 11% to 5%.

## 6. Conclusions

This study focuses on scientific process parameter setup and simulation as the main research themes. A scientific process parameter setup was developed based on nozzle pressure and screw position, with the process parameter search sequence being injection speed, *V*/*P* switchover position, packing pressure, and packing time. This scientific process parameter setup was applied to both experiments and simulations to identify the most suitable process parameters. Finally, the results from the experiments and simulations were compared, and efforts were made to correct the factors causing discrepancies between them. The following conclusions were drawn from this study:

(1) In the injection speed experiments, this study observed that when the injection speed was higher, the change in relative viscosity was smaller; conversely, when the injection speed was lower, the change in relative viscosity was greater.
(2) In the *V*/*P* switchover position experiments, by performing a second derivative on the nozzle pressure curve, the point of rapid pressure increase could be identified and correlated with the screw position, allowing for the determination of the screw position at which the cavity was fully filled.
(3) In the packing time experiments and simulation, this study found that by calculating the melt mass in front of the screw using the material’s *P*–*V*–*T* model and screw position, and by determining the difference in melt mass, a time point close to the gate solidification time could be identified.
(4) Through the injection speed simulation, this study observed that the relative viscosity showed a similar trend to the melt viscosity, demonstrating the feasibility of using this method to determine the appropriate injection speed.
(5) The simulation results for the *V*/*P* switchover position show that the nozzle pressure inflection point corresponded to the cavity reaching 100% fill. Therefore, the screw position identified by the pressure inflection point was the position where the cavity was fully filled.
(6) Identifying that the time constant influences the precision of the simulation and using it to minimize the simulation’s difference from the experiment can reduce the cost of machine model integration parameters (MMIPs).
(7) The calibrated injection speed simulation results show that by adjusting the time constant, the convergence of the simulated relative viscosity curve could be made more effective, making it more closely aligned with the experimental injection speed results.
(8) By applying the time constant to calibrate the injection speed and *V*/*P* switchover position in the simulation, the errors were significantly reduced by 14% and 6%, respectively, compared to the uncalibrated simulation. This study highlights the effectiveness of the CAE-based scientific process parameter setup in optimizing the injection-molding process.
(9) Through CAE and scientific molding trials, employees without extensive experience can follow this approach to achieve T0 mass production.

## Figures and Tables

**Table 1:** Volume and number of mesh elements of each component.

	Number of Solid Mesh Elements	Volume of Plastic (cc)
Plastic part	3,643,466	257.15
Runner	13,888	2.59
Nozzle zone	115,104	371.69
Mold base	2,913,702	-
Cooling channel	1,633,444	-
Total	8,319,988	631.43

**Table 2:** Injection speed experimental parameters.

Fixed parameters			
Melting temperature (°C)	215	Mold temperature (°C)	50
*V*/*P* switchover position (mm)	28	Clamping force (ton)	100
Packing time (s)	5	Cooling time (s)	40
Varying parameters			
Packing pressure (bar)	40, 35, 30, 25, 20, 15, 10, 5		
Injection speed (%/(mm/s))	30/48, 35/56, 40/64, 45/72, 50/80, 55/88, 60/96, 65/104, 70/112, 75/120, 80/128, 85/136, 90/144, 95/152, 100/160		

**Table 3:** *V*/*P* switchover position experimental parameters.

Fixed parameters			
Melting temperature (°C)	215	Mold temperature (°C)	50
Injection speed (%/(mm/s))	75/120	Packing pressure (bar)	5
Packing time (s)	5	Cooling time (s)	40
Clamping force (ton)	100		
Varying parameters			
*V*/*P* switchover position (mm)	21, 20, 19, 18, 17, 16, 15, 14		

**Table 4:** Packing pressure experimental parameters.

Fixed parameters			
Melting temperature (°C)	215	Mold temperature (°C)	50
Injection speed (%/(mm/s))	75/120	Packing time (s)	55
*V*/*P* switchover position (mm)	19	Cooling time (s)	5
Clamping force (ton)	100		
Varying parameters			
Packing pressure (bar)	0, 5, 10, 15, 20, 25, 30, 35, 40		

**Table 5:** Packing time experimental parameters.

Fixed parameters			
Melting temperature (°C)	215	Mold temperature (°C)	50
Injection speed (%/(mm/s))	75/120	Packing pressure (bar)	25
*V*/*P* switchover position (mm)	19	Clamping force (ton)	100
Varying parameters			
Cooling time (s)	42, 40, 38, 36, 34, 32, 30, 28, 26		
Storage delay time (s)	22, 20, 18, 16, 14, 12, 10, 8, 6		
Packing time (s)	13, 15, 17, 19, 21, 23, 25, 27, 29		

**Table 6:** Injection speed simulation parameters.

Fixed parameters			
Melting temperature (°C)	215	Mold temperature (°C)	50
*V*/*P* switchover position (mm)	28	Packing pressure (bar)	5
Packing time (s)	5	Cooling time (s)	40
Varying parameters			
Injection speed (%/(mm/s))	30/48, 35/56, 40/64, 45/72, 50/80, 55/88, 60/96, 65/104, 70/112, 75/120, 80/128, 85/136, 90/144, 95/152, 100/160		

**Table 7:** *V*/*P* switchover position simulation parameters.

Fixed parameters			
Melting temperature (°C)	215	Mold temperature (°C)	50
Injection speed (%/(mm/s))	90/144	Packing pressure (bar)	5
Packing time (s)	5	Cooling time (s)	40
Varying parameters			
*V*/*P* switchover position (mm)	21, 20, 19, 18, 17, 16, 15, 14		

**Table 8:** Packing pressure simulation parameters.

Fixed parameters			
Melting temperature (°C)	215	Mold temperature (°C)	50
Injection speed (%/(mm/s))	90/144	*V*/*P* switchover position (mm)	16
Packing time (s)	55	Cooling time (s)	5
Varying parameters			
Packing pressure (bar)	0, 5, 10, 15, 20, 25, 30, 35, 40		

**Table 9:** *V*/*P* switchover position simulation calibration parameters.

Fixed parameters			
Melting temperature (°C)	215	Mold temperature (°C)	50
Injection speed (%/(mm/s))	80/128	Packing pressure (bar)	5
Packing time (s)	5	Cooling time (s)	40
Time constant	0.23		
Varying parameters			
*V*/*P* switchover position (mm)	21, 20, 19, 18, 17, 16, 15, 14		

**Table 10:** Different *V*/*P* switchover positions corresponding to the pressure transition and volume fraction.

*V*/*P* switchover position (mm)	14	15	16	17	18	19	20	21
Screw position at pressure transition (mm)	16.2	16.8	16.9	19.4	29.7	29.5	29.7	29.7
Volume fraction (%)	100	100	99.92	97.52	88.57	88.86	88.86	88.86

## Data Availability

The data are not publicly available due to restrictions, e.g., their containing information that could compromise the privacy of the research participants.
